# Brickognize: Applying Photo-Realistic Image Synthesis for Lego Bricks Recognition with Limited Data

**DOI:** 10.3390/s23041898

**Published:** 2023-02-08

**Authors:** Joel Vidal, Guillem Vallicrosa, Robert Martí, Marc Barnada

**Affiliations:** 1 Computer Vision and Robotics Institute, University of Girona, 17003 Girona, Spain; 2 Tramacsoft GmbH, Schloßstraße 52, 60486 Frankfurt am Main, Germany

**Keywords:** photo-realistic rendering, image synthesis, limited data, object recognition, deep learning

## Abstract

During the last few years, supervised deep convolutional neural networks have become the state-of-the-art for image recognition tasks. Nevertheless, their performance is severely linked to the amount and quality of the training data. Acquiring and labeling data is a major challenge that limits their expansion to new applications, especially with limited data. Recognition of Lego bricks is a clear example of a real-world deep learning application that has been limited by the difficulties associated with data gathering and training. In this work, photo-realistic image synthesis and few-shot fine-tuning are proposed to overcome limited data in the context of Lego bricks recognition. Using synthetic images and a limited set of 20 real-world images from a controlled environment, the proposed system is evaluated on controlled and uncontrolled real-world testing datasets. Results show the good performance of the synthetically generated data and how limited data from a controlled domain can be successfully used for the few-shot fine-tuning of the synthetic training without a perceptible narrowing of its domain. Obtained results reach an AP50 value of 91.33% for uncontrolled scenarios and 98.7% for controlled ones.

## 1. Introduction

The latest advances in object detection show the potential of machine learning, and particularly of Convolutional Neural Networks (CNN), to expand automation efficiently solving a wider range of repetitive, tedious or dangerous tasks. The most successful approaches, based on supervised deep learning, have been used to solve ever-increasing challenging automation problems, especially with an increasing number of classes and wider scene domains [1,2,3]. Nevertheless, the efficacy of these techniques is still mostly bounded to the quantity and quality of the training data [4].

For real-world applications, data gathering and labeling commonly involve a challenging process and a tremendous effort that complicates the usage of deep learning techniques or limits their performance for most systems. In order to increase feasibility and reduce development time, some authors have proposed automatized data acquisition and labeling frameworks or the use of data from already available sources [5,6]. Nevertheless, these solutions are very task-specific and subject to strong environmental constraints that highly limit their real-world applicability. Therefore, at the end, most applications still require tremendously laborious and non-generalist labeling processes.

Manually searching and identifying Lego®-like bricks for classification represents a simple but monotonous and time-consuming process for a person. The large number of existing different bricks, the high similarity between them, their relatively small size and plain color patterns further adds to the tediousness of the job [7]. As such, the automation of the problem can be a clear benefit for commercial applications. Some examples of commercial applications are the identification of missing or extra bricks, verifying the completeness of sets, construction based on recommendations and automatic inventory for reselling. While manual image data acquisition and labeling of bricks is a solution to consider, especially for small subsets of data, it can easily become an unfeasible task that clearly does not scale well. The enormous amount of existing variety of bricks and the required effort for obtaining wide-domain data acquisition and annotation incur a rather huge amount of expensive manual work. Although the problem could be simplified for highly controlled environments, i.e., fixed background and single-brick classification, it would limit its scalability and applicability for high production environments and would still require a significant amount of work. In this regard, few related works have been proposed for Lego bricks recognition.

An existing commercial solution, Instabrick [8], proposed a hardware-based solution for keeping inventories and identifying single Lego pieces. The cloud-based system is based on a neural network trained with images acquired through the years among all the system users on a progressive training scheme.

Another existing project, named RebrickNet [9], proposed a multi-piece recognition network also progressively trained by manually taken images and videos of single Lego pieces with off-the-shelf cameras. The training data are gathered under a set of strong capturing constraints, e.g., fixed angle, fixed background, with the help of their online community. Although the project already collected a few images for each Lego part over several years, these were shown not to be sufficient, and they are now requesting 10-second videos of individual pieces to train the system.

In a different direction, the Brickit app [10] seems to show a much more flexible set-up, where the focus is not on the exhaustive recognition of all appearing bricks but on reliably identifying a subset of the bricks that can compose different complete models. Unfortunately, to the best of the authors’ knowledge, details of the employed training system have not been released by the company.

It can be noticed that in addition to the existing amount of Lego pieces, the continuous expansion of the product line, with new bricks released periodically [7], further increases the necessity of a flexible and scalable solution to the problem.

Based on these requirements, an ideal solution should adhere to the following:

- Not require manually annotated data or, alternatively, a very limited amount.
- Be scalable, flexible and automatic in order to be extended to all Lego bricks and sets, and even other items.
- Work on a wide domain of scenes, with different backgrounds and illuminations.

**Figure 1:**
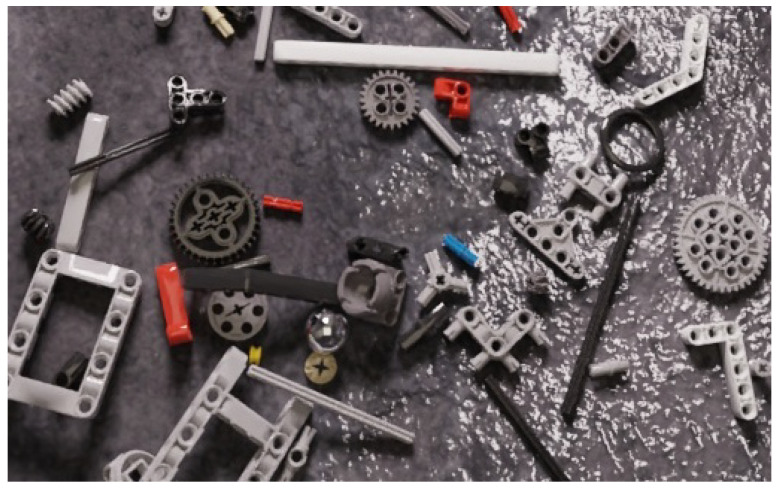
Synthetic image generated by the proposed rendering.

Synthetic data, obtained by rendering a computer simulation of real-world scenes, are a highly efficient and flexible way of obtaining training data, see Figure 1.

Recent advances in GPU hardware and 3D rendering algorithms provide a powerful setup for photo-realistic image synthesis. Although computer simulation is nowhere near capturing real-world fine details, it can effectively generate synthetic images with convincing scene details and light patterns. These so-called photo-realistic images have a high level of detail on light, physics and textures and can provide a strong base of perfectly annotated training data. Therefore, with high flexibility and scalability, synthetic data can be a tremendous cost-efficient solution to both acquisition and precise labeling, generating large training datasets at near no cost. In addition, it can also help to generate real-world unobserved outliers and pretrain complex systems such as semi-automatic labeling processes.

In this paper, an image synthesis solution is proposed for recognizing Lego-like bricks in a limited data scenario for a real-world application. Based on a Mask Regions with CNN (Mask R-CNN) deep learning model, a 3D photo-realistic rendering environment is proposed to substitute (and boost) real data. Initially, the training value of a limited set of manually annotated real data is evaluated for both random weight initialization and a transfer learning scheme using the COCO dataset [5]. These results are later compared with the results obtained from the proposed fully synthetic photo-realistic training. Finally, the real data are used for the few-shot fine-tuning of the synthetic training to help to compensate the synthetic-to-real gap. Therefore, we proposed the following steps:

- Synthetic photo-realistic data generation using the 3D CAD models of the bricks.
- Train a Mask R-CNN, pretrained with COCO, using the synthetic data.
- If real data are available, use few-shots fine-tuning on the synthetically trained network.

It is important to notice that the recognition of Lego bricks has several good conditions for this study. First, the Lego bricks are probably the most complete extensive collection of 3D CAD modeled real-world objects publicly available. Second, the recognition of lego bricks is challenging as there exist different objects sharing very similar features. Third, the problem is accessible by the broader public at a low budget. Fourth, the scalability of the solution is a key point of this problem.

Finally, the contributions of the presented solution can be summarized by the following points:

- Propose and evaluate a photo-realistic image synthesis approach to solve the shortcomings found on real-world applications with limited data.
- Present a leveraging set of state-of-the-art techniques to provide a cost and labor-efficient solution to the challenges of manual annotation while reaching top performance.
- Show how few-shots fine-tuning can help to fill the synthetic-to-reality gap without decreasing the working domain on real-world applications.
- Present a novel, scalable and automatic deep learning solution to Lego bricks detection and classification.
- Create and publicly release a dataset with semantic segmentation labels for real images of a Lego product in a *controlled* and *uncontrolled* environment.

The rest of the paper is organized as follows. Section 2 describes the network and learning techniques utilized. Section 3 describes the generation of synthetic data with annotations. Section 4 describes the different datasets used in learning, validation and testing. Section 5 describes the results obtained from the different tests and discusses them. Finally, in Section 6, conclusions are presented.

## 2. Recognition Method

**Figure 2:**
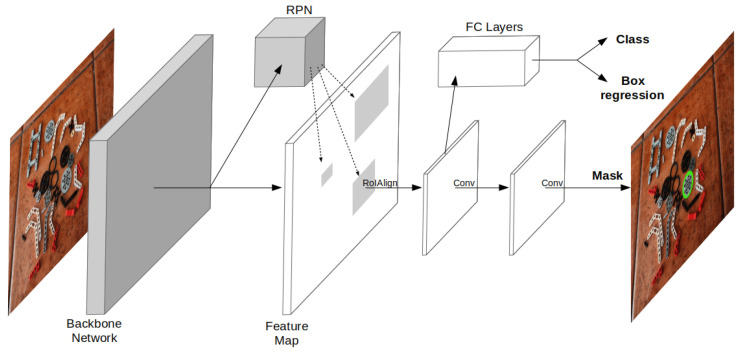
Architecture of the Mask R-CNN framework.

Supervised deep learning solutions based on CNN were first applied solely to the classification problem [11]. Based on these classification networks, Girshick et al. [12] presented Regions with CNN (R-CNN) features, extending the concept to detection by employing a *recognition using regions* paradigm. The detection is achieved by individually classifying candidate regions given by a category-independent region proposal method, i.e., selective search. In detail, the solution used a pretrained CNN to extract features of the fixed-size affine wrapped candidate regions, which were classified using category-specific linear SVMs. The method was later improved by Girshick [13], in a variant named Fast R-CNN, for which a single feature image is generated from the source image and fixed-size feature vectors are obtained by max pooling each region candidate. Then, each feature-vector is fed into a sequence of fully connected layers that output both a soft-max probability estimate and a refined bounding-box. Commonly, the feature extraction network is known as the backbone, and the output network is known as the head. Ren et al. [14] further improved the method, named Faster R-CNN, by replacing the classical greedy region proposal methods with a new Regional Proposal Network (RPN) that outputs region proposals directly from the feature image. Finally, He et al. [15] proposed a Mask R-CNN variant, extending the Faster R-CNN network head to output a binary mask for each region; see Figure 2.

In this paper, a Mask R-CNN network is used, consisting of a ResNet-50 [16] backbone with Feature Pyramid Network (FPN) alongside a RPN head. A batch-size of 2 and a Stochastic Gradient Descent (SGD) optimizer [17] with a $10^{-3}$ learning rate and 0.9 momentum are used.

## 3. Synthetic Data Generation

Synthetic data are generated with Blender [18], an open-source 3D modeling and rendering packages, through BlenderProc [19]. BlenderProc is a modular procedural pipeline that runs within the Blender’s environment, providing a set of tools for the semi-automatic procedural generation of scenes. The provided modules include tools for data loading, camera definition, scene characterization, illumination, physics-based object placement, rendering and data labeling. The modular nature of the pipeline allows the development of additional tool-specific functionalities. Once a scene is constructed, photo-realistic rendering is achieved using Blender’s physically-based path tracer for production rendering, named *cycles*.

**Figure 3:**
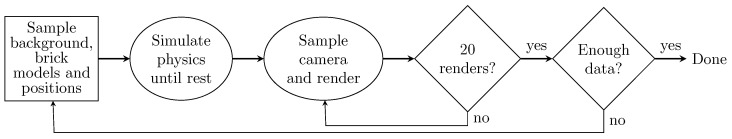
Synthetic data generation flowchart.

In this work, a standard scene was constructed as a square room with a ceiling plane acting as a light emission shader. Different random scenes were generated by randomly changing the camera positions, bricks placement, background and light position. In detail, bricks were uniformly sampled, for each scene, on a 3D region above the floor plane with different initial poses and physically located on the floor by the simulation of gravity. As floor textures, Physically Based Rendering (PBR) textures were used, representing a wide range of backgrounds, including textures found in nature, house-hold and industrial environments. A point light was also randomly set to different positions, levels of intensity and colors. Finally, each scene was rendered from 20 different random camera locations following a spherical shell distribution around the center of the room. This process is repeated until enough synthetic data have been generated (Figure 3). Figure 1 shows an example of a synthetic rendering.

Finally, for labeling, an additional flat single-color per object instance rendering is performed to generate a segmentation map. This segmentation map is in turn post-processed to extract an object annotation for each color, following the COCO dataset format [5], which is supported by most deep learning frameworks.

## 4. Datasets

For this work, two datasets of Lego pieces are used: a synthetic dataset and a real dataset. The synthetic dataset, generated by the method described in Section 3, is only used for training. The real dataset, created manually with camera images, includes two different sets of data, named *controlled* and *uncontrolled*, and is split into training, validation and testing. The testing dataset has been made publicly available (www.tramacsoft.com/brickognize, accessed on 18 December 2022). Both datasets consist of annotated RGB images with a variable amount of 76 distinct Lego bricks (Table 1) located on top of a planar surface (all the used data were captured and annotated by the authors).

The *controlled* training data (20 images) and *controlled* validation data (5 images) would be the only required datasets to be manually annotated in the case of a real-world application. This paper, however, also includes the *uncontrolled* data, to test the approach against a much broader domain than the provided training images, and the *controlled* test data, to compare the broad application of the model to a more concrete case that could be applicable to industry.

### 4.1. Training Data

Two different datasets are provided for training.

#### 4.1.1. Real

**Figure 4:**
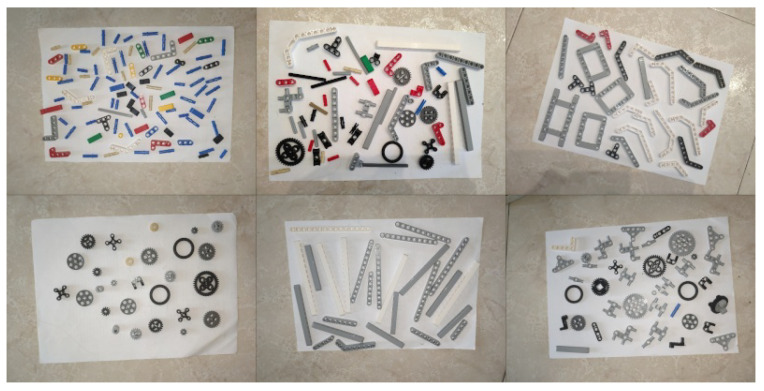
*Real* dataset with real-world images taken perpendicularly, with controlled light and fixed white background.

The real dataset is an easy-to-annotate 20 real-world images dataset with controlled lighting and camera angles, designed for fast acquisition and labeling. Images were taken perpendicularly on a fixed white background. The dataset was acquired with a standard cellphone camera and manually annotated (Figure 4). A non-occlusion criterion was forced in order to simplify the hand-made labeling process.

#### 4.1.2. Synthetic

**Figure 5:**
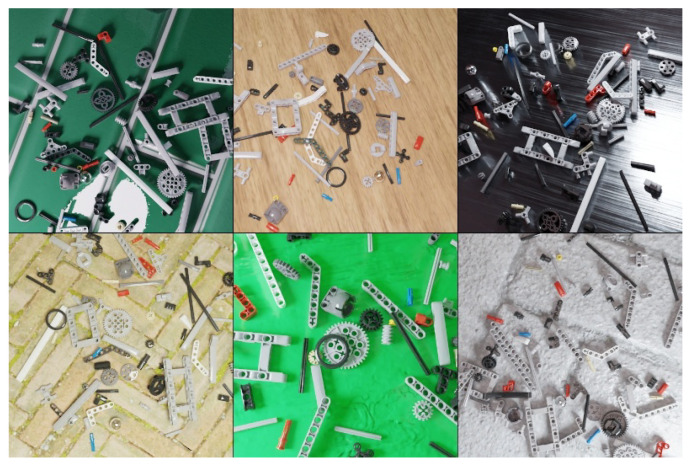
*Synthetic* dataset, showing synthetically generated images with different backgrounds and camera and light positions.

The synthetic dataset was generated with the synthetic data generation process described in Section 3 using the 3D CAD models of the bricks (Figure 5). The training dataset includes 1*K* different scenes with 20 images per scene, summing up to more than 20,000 rendered images. All the images were rendered with a size of 512 × 512. Overall, the dataset has more than 1.1 million annotated items.

### 4.2. Validation Data

A five-images dataset is provided for validation, taken in the same conditions as the *real* training dataset.

### 4.3. Testing Data

Two different datasets are provided for testing.

#### 4.3.1. Uncontrolled

**Figure 6:**
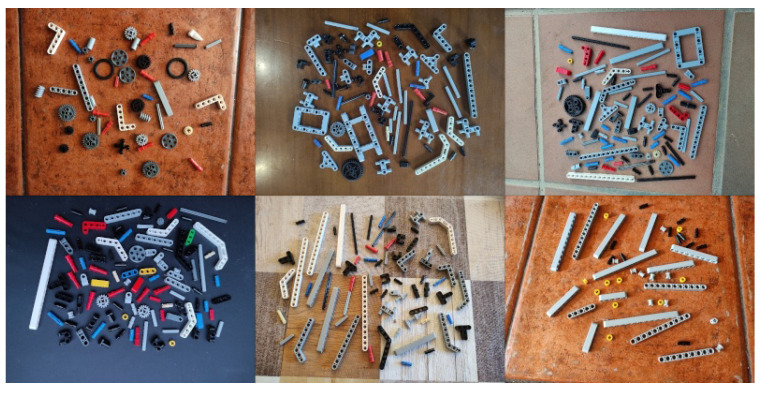
*Uncontrolled* dataset with real images and different backgrounds with different camera positions and illuminations.

This set is focused on a real-world broad testing scenario, where training is done on a single domain but usage is not constrained to other domains. It consists of 100 images with 6700 annotations collected with 5 different randomly chosen indoor and outdoor household scenes (Figure 6), with different camera positions and illuminations, acquired with a conventional smartphone camera and manually annotated.

#### 4.3.2. Controlled

This set is focused on real-world industrial application, where training and testing are done on a single domain. It consists of 160 images acquired with the same setup as the *controlled* training data.

## 5. Experiments and Results

**Figure 7:**
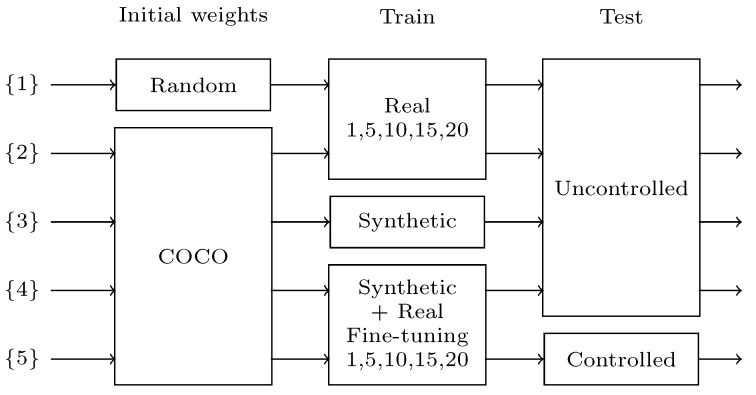
Schematic of differences between test results.

In this section, several experiments are performed to study the advantages of using synthetic data generation and also few-shot fine-tuning with real data to overcome the limited data problem (Figure 7). First, in **{1}**, the available limited data are trained with a random weights initialization. Then, in **{2}**, the network is initialized with the well-known COCO dataset [5]. Both experiments include the evaluation of different numbers of training images: 1, 5, 10, 15 and 20. These two experiments establish the base of what is achievable using only limited real data.

Then, the proposed synthetic training data generation is used on **{3}** showing a huge performance increase. Finally, few-shot fine-tuning is used on top of the synthetic training, testing the results on two different domains for a final product. In **{4}**, the network is tested against a broader domain than the real labeled data used for training, showing the results of a generalist application fine-tuned with data from a controlled domain. In **{5}**, the tests are conducted against the same controlled domain as the training data, showing the results of a dedicated controlled application, similar to what could be applied in industrial environments. Those two last experiments also evaluate the effects of the number of real training images used for fine-tuning.

All networks are trained for 20 epochs, and a validation-based early stopping is used. For validation, the *controlled* validation data are used with the *segm-AP50* metric (see Section 5.1.1) for all trainings. When using few-shot fine-tuning, the network is first trained on the *synthetic* dataset and then on the *controlled* training data.

### 5.1. Evaluation Metrics

The trained networks are evaluated using two different metrics: a more standard recognition metric and an application-oriented metric.

#### 5.1.1. Segm-Ap50

Segm-AP50 is a well-known metric used to evaluate the performance of an object detector. It is widely used in PASCAL-VOC [6] and COCO [5] challenges. The metric computes the area under the precision-recall curve, counting detections as true positives when the Intersection over Union (IoU) is larger than 50%. The results are averaged over all classes to provide the final value of the metric.

#### 5.1.2. Application-Oriented Metrics

**Figure 8:**
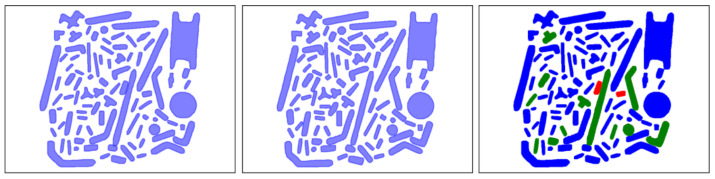
(**left**) Ground truth annotations for an image. (**center**) Detections from the trained network. (**right**) Correctly detected and classified bricks in blue (76 bricks), *not detected* bricks in red (2 bricks, 2.2%) and *misclassified* in green (12 bricks, 13.3%). The obtained application-oriented metric score is 84.5%.

This metric is defined by the authors of this paper to account for the expected result of a brick-detection application, which produces a brick classification probability for each image region. To compute this metric, the first step is to apply a non-maximal suppression of the detections produced by the network. For each pair of detections that overlap each other by more than 10% of the area of the smallest one, only the one with the highest detection score is kept. After the non-maximal suppression, each detection is matched against the annotated ground truth (Figure 8). For the comparison with the ground truth, the detection with the largest overlap area (minimum of 10%) is associated with it. If the predicted class is the same as the ground truth class, then the brick is correctly classified. If the class is different, the prediction is considered *misclassified*. If no overlap is found for a ground truth annotation, it is considered as *not detected*. The score is computed as the total number of ground truth annotations minus both the detection and classification errors divided by the total number of ground truth annotations (Figure 8 for a detection example).

### 5.2. Results

#### 5.2.1. Standard Training with Limited Data

Initially, the Mask R-CNN network is trained using the limited *controlled* training data and tested against the *uncontrolled* testing data. This evaluation shows the baseline performance of a standard method for extreme limited data cases on highly uncontrolled real-world scenarios. The training is performed using both initial random weights **{1}** and transfer learning with pretrained weights **{2}** from the COCO dataset [5].

Looking at the segm-AP50 metric results (Table 2), it is clear that both networks fail to provide any useful result with the limited training data, even when using transfer learning. Therefore, they do not show any application-oriented metric value.

#### 5.2.2. Training Using Synthetic Data

As seen before, limited training data have almost no value on a standard supervised training scheme. Here, these real-data training results are compared against a Mask R-CNN network solely trained on the *synthetic* training data **{3}**. Both networks are again tested on the *uncontrolled* dataset with real-world scenarios.

The obtained results clearly show the tremendous value of the proposed synthetic data generation (Table 3), reaching a segm-AP50 value of 83.3%, improving significantly over the results obtained with standard training with the limited data, whose results were 4.25% for the best case with 20 images.

Looking at the application-oriented metric results (Table 4), the much higher value of the synthetic solution is again validated with a 82.1% score, against the much lower 22.58% for the real training data. In depth, the not detected and misclassified results show the incapacity of the COCO+20-Real network to learn enough information to detect most pieces and also classify them properly. As suspected, for cases with limitation in training data, a synthetic solution can provide an alternative solution at virtually no cost.

#### 5.2.3. Few-Shot Fine-Tuning of Synthetic Training

In order to improve the results of the synthetic training, a few-shot fine-tuning is proposed **{4}**. The main idea is to use the available limited real data to help fill the synthetic-to-real domain gap, even though the training data were acquired on a different domain, i.e., a partially controlled environment.

The obtained results show that for the larger number of shots used for fine-tuning, the results obtained in both segm-AP50 and application metric increase rapidly, showing a saturation behavior at around 20 shots (Table 5 and Table 6). Results improve significantly with respect to the purely synthetic ones, closing the synthetic-to-real gap with a minimum effort in terms of manual annotation.

#### 5.2.4. Performance on a Partially Controlled Environment

Using 20 shots fine-tuning on top of a synthetic training, the system reaches a performance of 91.33% for segm-AP50 and 91.21% for the application metric, for the highly uncontrolled test scenario of the *uncontrolled* test dataset. Although this performance is high for most generalist applications, it may not be enough for other solutions that require more exhaustive detections, such as industrial applications. In this direction, the Synthetic+20-Real is tested against the *controlled* test data **{5}**.

The obtained results show again that the more images are used for fine-tuning, the better the results obtained in both segm-AP50 and application metric (Table 7 and Table 8). In detail, although the results start at a lower score with respect to the previous testing against the *uncontrolled* dataset, the scores improve rapidly and improve significantly, after only five images, over the synthetic results. Finally, they show almost perfect results against the *controlled* test dataset, with the same manual annotation effort required in the previous results.

This shows that good results can be obtained by leveraging the power of synthetic data generation that is applied either to a generalist application with good results or to a more specialized one with significantly better results.

### 5.3. Discussion

**Figure 9:**
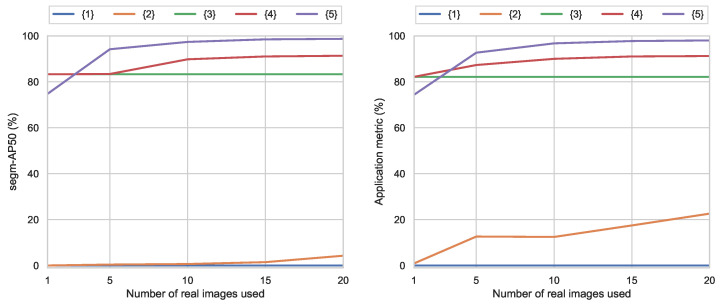
Overall results of the different tests with segm-AP50 and application metrics.

Overall, the obtained results show the great value of the proposed image synthesis for practical cases with limited data and also how limited data can be used to boost performance with a few-shot fine-tuning (Figure 9). This study has focused on using a maximum of 20 training images, based on scalability and feasibility considerations of practical real-world applications. However, if a larger amount of images is considered (240 training and 80 validation images), preliminary testing results still show segm-AP50 values substantially lower (56.9%) for real data than synthetic training. Therefore, purely synthetic (83.3%) and the few-shot fine-tuning (91.33%) still clearly exceed the results of training datasets up to 10 times larger. In the same direction, results for few-shot fine-tuning saturate for more than 20 shots, improving less than 1% even using 10 times more images. These results also show that fine-tuning with real data from a fixed controlled domain improves results for the uncontrolled scenes, effectively reducing the synthetic-to-real gap, without the unwanted effect of reducing the scene domain.

Analyzing the errors of the proposed system in more detail, most of the *not detected* errors are on bricks that are touching each other on the image and for which it is difficult to discern one from the other, specifically when they are of the same color and their boundaries are not clearly distinguishable. Most of the *misclassified* errors are between bricks that are almost identical from a certain point of view or have the same shape but can have different lengths; even for a human annotator, these are difficult to differentiate without using other bricks in the image to have a sense of scale between them.

Compared with existing solutions, the presented approach shows clear advantages. On the one hand, the proposed method has the ability to be trained with synthetic data, reaching high performance scores on uncontrolled environments using a scalable and effortless data generation process. This point clearly improves upon past approaches, such as Instabrick [8] and RebrickNet [9], that require a larger amount of time and effort to obtain training data. On the other hand, the presented method can also be fine-tuned with real data to obtain higher performance without reducing its working domain. Compared with other methods, the proposed few-shot fine-tuning process only requires a much smaller and limited set of less than 20 images, which can be captured in a controlled environment specially designed to simplify the task.

The paper has focused on what could be regarded as a very specific problem: the detection and classification of Lego bricks. However, no assumptions on the type of objects, data and acquisition scenarios have been established, for which the overall framework, results and benefits of using synthetic with limited annotated data could be extrapolated to other applications, particularly for industry and quality control.

For the particular application of Lego brick detection and classification of this paper, the annotation of each real image took an average of 50 min using a dedicated software. This further illustrates the value and cost-efficiency of using synthetic data, which is automatically annotated, compared to real world annotations.

Finally, these are the main limitations found:

- Not all objects can be easily rendered; non-rigid objects such as chains, transparent materials, flexible objects made of rubber.
- Challenging cases for recognition are axes of similar lengths and identical-looking objects from specific view points.
- Although the synthetic-to-real gap was reduced by means of the real data few-shots fine-tuning, there is still need for real-world data, and the problem is not yet fully solved, which could be a challenge for very highly demanding tasks.

## 6. Conclusions

A framework for photo-realistic data rendering has been proposed to generate realistic synthetic data for the task of recognizing Lego-like bricks to overcome the problem of limited available annotated real data.

A deep learning recognition method based on Mask R-CNN and trained with different number of real images with and without a pre-training on synthetic images is tested against a generalist real-wold dataset.

It has been shown that, when trained with the limited real images, although the transfer learning method outperforms the one with random weights, both methods achieve no usable results. In addition, the benefits of using synthetically generated images have been shown, obtaining results that clearly outperform the real data-only-trained network at virtually no cost for data acquisition. Finally, the combination of synthetic with real data in order to close the synthetic-to-real gap has been shown to obtain the best results, needing only 10 images to overpass 90% in the segm-AP50 metric.

For more industry-oriented applications, it has also been shown that, when testing against the same domain as the limited training data, the combination of real and synthetic data also improves the performance, achieving 98.7% segm-AP50, providing competitive results with a small effort on the annotation side.

## Figures and Tables

**Table 1:** Set of 76 different bricks used in this work (bricks and colors are identified by their Lego identification).

Brick	Color	Brick	Color	Brick	Color	Brick	Color	Brick	Color
2780	0	3707	0	44809	4	32525	71	63869	71
2815	0	3708	0	59443	4	32526	71	64178	71
32013	0	3737	0	32123b	14	3649	71	64179	71
32014	0	41678	0	32523	14	3673	71	6536	71
32034	0	45590	0	32009	15	3713	71	87082	71
32072	0	60483	0	32278	15	4019	71	99773	71
32184	0	60484	0	32348	15	40490	71	10928	72
32269	0	6629	0	32526	15	41239	71	3648b	72
32270	0	32523	1	32556	19	44294	71	4185	72
32291	0	43093	1	3749	19	4519	71	42003	72
32449	0	6558	1	6589	19	4716	71	55013	72
32498	0	32523	2	6587	28	48989	71	87083	72
32523	0	32054	4	32073	71	55615	71		
33299a	0	32062	4	32271	71	57585	71		
3705	0	32140	4	32316	71	60485	71		
3706	0	32523	4	32524	71	62462	71		

**Table 2:** Segm-AP50 results, comparing **{1}** and **{2}**.

Training Images	1	5	10	15	20
**{1}** Real	0.0%	0.0%	0.0%	0.0%	0.0%
**{2}** COCO+Real	0.0%	0.40%	0.64%	1.45%	4.25%

**Table 3:** Segm-AP50 results comparing **{2}** and **{3}**.

	Segm-AP50
**{2}** COCO+20-Real	4.25%
**{3}** Synthetic	83.3%

**Table 4:** Application-oriented metric results, comparing **{2}** and **{3}**.

	Not Detected	Misclassified	Score
**{2}** COCO+20-Real	31.76%	45.66%	22.58%
**{3}** Synthetic	4.3%	13.6%	82.1%

**Table 5:** Segm-AP50 results comparing **{3}** with few-shot fine-tuning against the *uncontrolled* dataset **{4}**.

n-shots	(Synthetic)	1-Real	5-Real	10-Real	15-Real	20-Real
Segm-AP50	83.3%	83.26%	88.39%	89.79%	91.03%	91.33%

**Table 6:** Application-oriented metric results comparing **{3}** with few-shot fine-tuning against the *uncontrolled* dataset **{4}**.

n-Shots	Not Detected	Misclassified	Score
Synthetic	4.30%	13.60%	82.10%
Synthetic+1-Real	4.30%	13.55%	82.15%
Synthetic+5-Real	3.97%	8.73%	87.30%
Synthetic+10-Real	2.92%	7.09%	89.99%
Synthetic+15-Real	2.66%	6.31%	91.03%
Synthetic+20-Real	2.58%	6.21%	91.21%

**Table 7:** Segm-AP50 results comparing purely-synthetic training with few-shot fine-tuning against the *controlled* dataset **{5}**.

n-shots	(Synthetic)	1-Real	5-Real	10-Real	15-Real	20-Real
Segm-AP50	74.77%	74.77%	94.17%	97.36%	98.48%	98.7%

**Table 8:** Application-oriented metric results comparing purely-synthetic training with few-shot fine-tuning against the *controlled* dataset **{5}**.

n-Shots	Not Detected	Misclassified	Score
Synthetic	0.88%	24.75%	74.37%
Synthetic+1-Real	0.88%	24.77%	74.35%
Synthetic+5-Real	0.23%	7.10%	92.67%
Synthetic+10-Real	0.10%	3.17%	96.73%
Synthetic+15-Real	0.06%	2.20%	97.74%
Synthetic+20-Real	0.04%	1.96%	98.00%

## Data Availability

Data available in a publicly accessible repository that does not issue DOIs. Testing dataset available at www.tramacsoft.com/brickognize.

## Abbreviations

The following abbreviations are used in this manuscript:

AP	Average Precision
CNN	Convolutional Neural Networks
COCO	Common Objects in Context
FPN	Feature Pyramid Network
GPU	Graphics Processing Unit
IoU	Intersection over Union
PBR	Physically Based Rendering
R-CNN	Regions with CNN
RGB	Red Green Blue
RPN	Regional Proposal Network
SVM	Support Vector Machine
VOC	Visual Object Classes
